# Therapeutic uses and applications of bovine lactoferrin in aquatic animal medicine: an overview

**DOI:** 10.1007/s11259-022-10060-3

**Published:** 2023-01-20

**Authors:** Sameh A. Abdelnour, Shakira Ghazanfar, Mahmoud Abdel-Hamid, Hany M.R. Abdel-Latif, Zhaowei Zhang, Mohammed A.E. Naiel

**Affiliations:** 1 Department of Animal Production, Faculty of Agriculture, Zagazig University, 44519 Zagazig, Egypt; 2 National Institute for Genomics Advanced and Biotechnology (NIGAB), National Agricultural Research Centre, Park Road, 45500 Islamabad, Pakistan; 3 Dairy Science Department, Faculty of Agriculture, Cairo University, 12613 Giza, Egypt; 4 Department of Poultry and Fish Diseases, Faculty of Veterinary Medicine, Alexandria University, 22758 Alexandria, Egypt; 5 National Reference Laboratory for Agricultural Testing (Biotoxin), Key Laboratory of Biology and Genetic Improvement of Oil Crops, Key Laboratory of Detection for Mycotoxins, Ministry of Agriculture and Rural Affairs, Oil Crops Research Institute of Chinese Academy of Agricultural Sciences, 430062 Wuhan, PR China

**Keywords:** Antioxidant, Diseases, Fish, Health benefits, Immunity, Lactoferrin

## Abstract

Aquaculture is an important food sector throughout the globe because of its importance in ensuring the availability of nutritious and safe food for human beings. In recent years, this sector has been challenged with several obstacles especially the emergence of infectious disease outbreaks. Various treatment and control aspects, including antibiotics, antiseptics, and other anti-microbial agents, have been used to treat farmed fish and shrimp against diseases. Nonetheless, these medications have been prohibited and banned in many countries because of the development of antimicrobial-resistant bacterial strains, the accumulation of residues in the flesh of farmed fish and shrimp, and their environmental threats to aquatic ecosystems. Therefore, scientists and researchers have concentrated their research on finding natural and safe products to control disease outbreaks. From these natural products, bovine lactoferrin can be utilized as a functional feed supplement. Bovine lactoferrin is a multi-functional glycoprotein applied in various industries, like food preservation, and numerous medications, due to its non-toxic and ecological features. Recent research has proposed multiple advantages and benefits of using bovine lactoferrin in aquaculture. Reports showed its potential ability to enhance growth, reduce mortalities, regulate iron metabolism, decrease disease outbreaks, stimulate the antioxidant defense system, and recuperate the overall health conditions of the treated fish and shrimp. Besides, bovine lactoferrin can be considered as a safe antibiotic alternative and a unique therapeutic agent to decrease the negative impacts of infectious diseases. These features can be attributed to its well-known antibacterial, anti-parasitic, anti-inflammatory, immunostimulatory, and antioxidant capabilities. This literature review will highlight the implications of bovine lactoferrin in aquaculture, particularly highlighting its therapeutic features and ability to promote immunological defensive pathways in fish. The information included in this article would be valuable for further research studies to improve aquaculture’s sustainability and the functionality of aquafeeds.

## Introduction

Antibiotics control infectious bacterial diseases in aquaculture; however, their extensive overuse will result in numerous unfavorable side effects, like the appearance of antibiotic-tolerant strains and leaving remains in the aquatic environments (Founou et al. 2016; Manyi-Loh et al. 2018; Abdel-Latif et al. 2020). Consequently, there is a pressing necessity to uncover new antibiotic replacements to be used in aquaculture to improve the disease resistance of farmed fish and shrimp (Peterson and Kaur 2018; Abdel-Tawwab et al. 2022). Several feed additives used as immunostimulants can stimulate the fish’s immune responses (Abdel-Latif et al. 2022a, b; Alagawany et al. 2021). In the aquaculture field, various immunostimulants have been examined in aquatic studies, such as chitin, *β*-glucans, phytochemical molecules, herbal immunomodulators, and several others (Ahmadifar et al. 2021; Farag et al. 2021), with proven immune-enhancing roles. However, researchers and aquatic scientists are still exploring new and effective alternatives with potent immune-stimulatory effects.

Milk has significant amounts and many active molecules, such as lactoferrins. Lactoferrin (LF) is a glycoprotein connected with plasma iron-transport protein transferrin (Adlerova et al. 2008). It contains a single peptide chain with two globular lobes, each comprising one iron-binding site (González-Chávez et al. 2009). Several reports indicate its ability for use as an immunostimulant with several other biological activities (Gifford et al. 2005). Moreover, it can boost the non-specific immune system and augment the resistance against many diseases in many fish and shellfish species (Moreno-Expósito et al. 2018; Yokoyama et al. 2019). Bovine milk is one of the common supplies of bovine lactoferrin (BLF), which has been used in several industrial uses. Numerous biological properties have been accredited to the functions of BLF, such as its antioxidant activity (Sandomirsky et al. 2003), an iron absorption, and anti-microbial activities (Bellamy et al. 1992). Besides, it possesses anti-fungal, antiviral, anti-parasitic, and anti-inflammatory properties (Trybek et al. 2016). Therefore, BLF may induce effective defense against different fungal, viral, and bacterial strains that may affect many aquatic animals.

In aquaculture, BLF possesses several beneficial effects (Luna-Castro et al. 2022). For instance, earlier reports showed that BLF could be used in fish diets to enhance resistance against several bacterial diseases caused by several bacterial strains such as *Aeromonas hydrophilia* in Asian catfish (*Clarias batrachus*) (Kumari et al. 2003) and *Streptococcus* species and *Vibrio anguillarum* in rainbow trout (*Oncorhynchus mykiss*) (Sakai et al. 1993). BLF can also improve the growth indices and stress tolerance in different fish species as goldfish (*Carassius auratus*) and Japanese flounder (*Paralychthys olivaceus*) (Kakuta 1996, 1998; Yokoyama et al. 2006), and enhance the immune responses (Anderson 1992), in various aquatic species like Asian catfish (Kumari et al. 2003), Siberian sturgeon (Eslamloo et al. 2012), and rainbow trout (Rahimnejad et al. 2012). Therefore, applying BLF in aquaculture nutrition to enhance the fish’s immune status is relatively important to ensure antibiotic-free aquaculture (Yokoyama et al. 2019; Morshedi et al. 2020). Nonetheless, the use of BLF in nutritional strategies may be affected by factors such as fish species, dose, culture system, diet, environmental conditions, and administration method (Fernandes and Carter 2017).

The recently published article by Luna-Castro and coauthors has focused on the effectiveness of BLF in the modulation of immunity, stress conditions, and bacterial disease resistance in aquaculture (Luna-Castro et al. 2022). Herein the present context, we will present an updated overview on the characterization, bioavailability, metabolism, absorption, and delivery of BLF. Moreover, we will spotlight the potential impacts of the inclusion of BLF in fish nutrition with special emphasis on growth, digestive enzymes, and intestinal epithelial health. The biological functions of BLF, as antibacterial, antioxidant, anti-inflammatory, anti-parasitic, and immunomodulatory effects, were also described. The information included in this article would be valuable for further research studies to improve the sustainability of aquaculture.

## Lactoferrin structure and resources

Lactoferrin (LF) is an 80 kD glycoprotein obtained from human and cow milks and their byproducts (Superti 2020). Colostrum has around seven times the LF found in the latter-produced milk (Villavicencio et al. 2017). LF may be present in fluids of various tissues and organs such as the eye, nose, respiratory tract, gastric tract, and others (Lönnerdal et al. 2020). Generally, it is widely released from mucosal surfaces and plays important functions in innate immune responses (Franco et al. 2018). It is produced via the epithelial cells in the udder (mammary glands) of cows and is directly secreted into milk (Nakajima et al. 2008). Moreover, prolactin modulates the amount of LF produced in the mammary glands (García-Montoya et al. 2012).

BLF has two homologous lobes (N and C) or four domains (N1 and N2, C1 and C2), with each lobe binding one ferric iron (Fe^3+^) (Baker and Baker 2009; Bokkhim et al. 2013). BLF structure enables it to transmit iron to the entire cells and control the quantity of free iron in the blood and extracellular secretions (Sinha et al. 2013). Iron transport regulation in fish is crucial in oxygen transport and cellular respiration (Krewulak and Vogel 2008). In addition, BLF can be linked to other minerals such as Zn^2+^, Mn^3+^, Cu^2+^, and Ce^4+^ (Soboleva et al. 2019). Specifically, the iron or other ions linked with BLF might be detached at low pH levels (pH < 4) (Bokkhim et al. 2013). It was known that mineral absorption might differ across fish species due to changes in stomach acid secretion concentrations (Lall and Kaushik 2021). Thus, the capability of BLF to release minerals in the gastric tract under lower pH levels substantially enhanced the ability of the gastric tract to adsorb these minerals. Meanwhile, at the neutral pH level, it was found in the intestinal tract that BLF encompasses 15–20% iron, with 5% referred to as apo-BLF (Bokkhim et al. 2013).

**Fig. 1:**
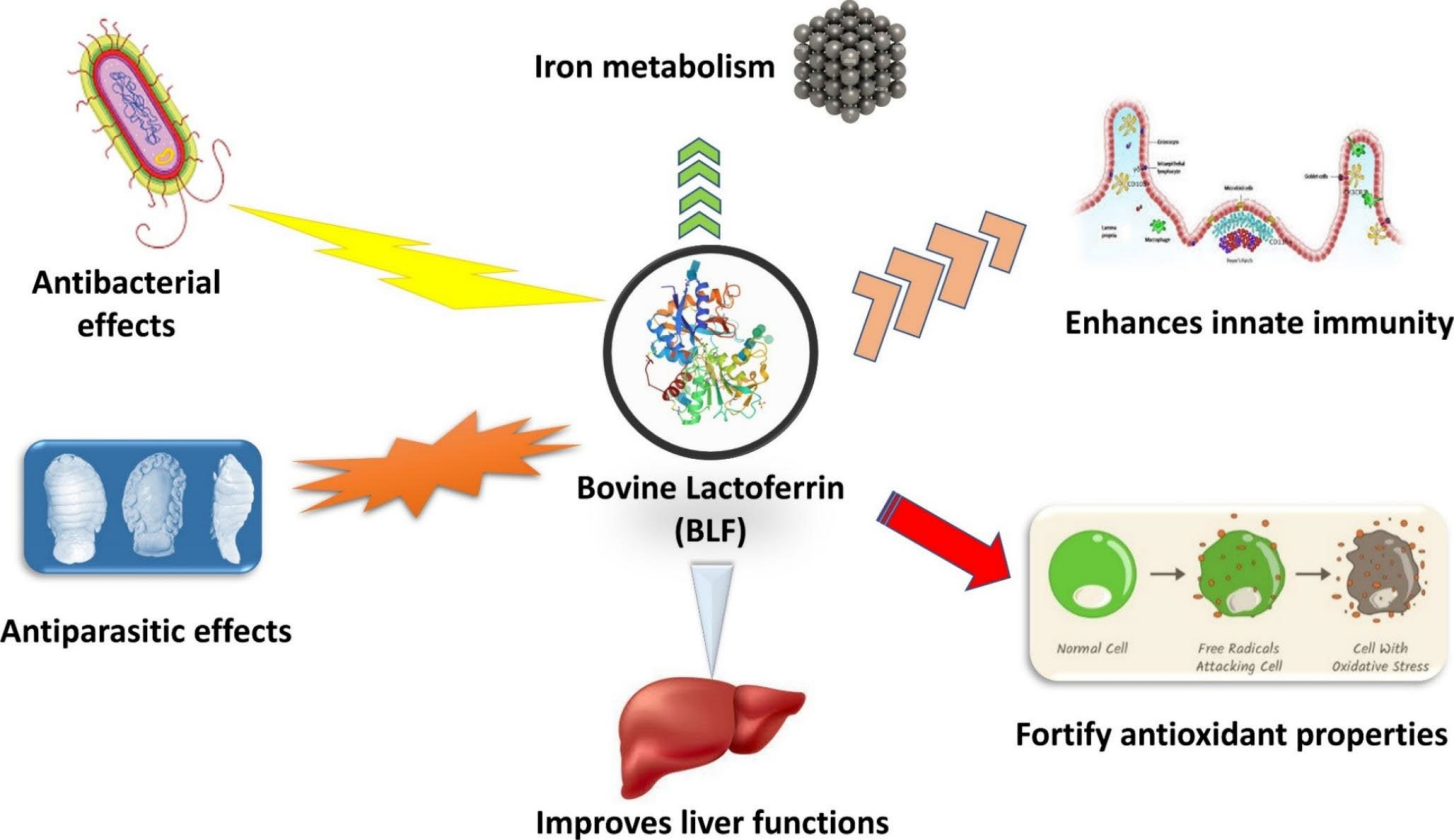
The general main biological activities of bovine lactoferrin (BLF)

The structure and chemical characteristics of BLF may be altered by iron binding (Bokkhim et al. 2013). LF1-11 (25 residues) and lactoferrampin (265–284 position) are the main functional peptides derived from BLF after stomach digestion (Hao et al. 2018). Other biologically active cationic-based peptides are found at locations 20–30, 17–31, 17–27, and 20–25 (Bokkhim et al. 2013; Hao et al. 2018). At the same time, lactoferrampin (265–284) and lactoferricin (17– 30) peptides were discovered to be more constant to ionic strength and to have more bactericidal activities (Baker and Baker 2009). The antibacterial properties of BLF may be related to the presence of these cationic peptides (Sinha et al. 2013). The general biological properties of BLF are described in Fig. 1, and its function will be investigated in more depth throughout the text.

## Bioavailability, absorption, biological mechanisms, and metabolism of BLF

In mammals, BLF can be easily absorbed into the bloodstream and digested in the gastrointestinal tract (GIT) through bile secretion, reaching a high peak value 12 h after oral administration (Harada et al. 1999). In human beings, dietary LF may quickly interact with iron and reach mucus and fluids, resulting in increased microbiostatic activity (Sharma et al. 2017). The orally given BLF will be extensively degraded into small molecules when it passes the GIT (Moreno-Expósito et al. 2018). Meanwhile, several functions of BLF are highly supported by protein structure integrity, and its digestion in the GIT induces damage to numerous of these features (Baker and Baker 2009). To produce bioactive fragments and perform their benefits as part of a diet, LF must be protected against GIT disorders (Superti 2020). While in aquaculture, depending on the inclusion level of BLF in the fish diets, it has been revealed that dietary BLF stimulates the development and proliferation of the intestinal epithelial cells (enterocytes) (Buccigrossi et al. 2007). Lately, numerous research studies have concentrated on improving LF’s oral bioavailability (Elzoghby et al. 2020), where the formulation of BLF delivery structures has been contacted with various molecules. The generally applied approaches to guard BLF throughout the oral passage and gastric digestion phases depend on the following features: (a) iron saturation, (b) PEGylation microencapsulation and (c) absorption enhancers (Yao et al. 2013, 2015). Recently, with the excessively growing of nanotechnology uses in different fields, the microencapsulation method is frequently applied to act as a shelter for BLF from the digestion process by protease enzyme in the GIT. Besides the micro-encapsulation with carbohydrates or proteins, liposomes can also help to avoid BLF gastric degradation (Liu et al. 2013).

As for absorption stimulators, numerous moleculescan transportthe BLF through the biological membranes. For instance, the chitosan molecule has been described to boost the adsorption of BLF via the intestinal cavity by opening the intercellular junctions. Even though chitosan or its derivatives are poorly soluble in acidic stomach pH, chitosan has been broadly applied for a range of cell delivery purposes (Yao et al. 2013). Until now, PEGylation and microencapsulation seem to be the main effectual methods for delivering higher BLF levels to the intestinal absorption sites.

Mucus is a sticky, slippery substance that coats the epithelial surfaces of fish. The mucus consists of anti-microbial enzymes, proteins, and water, making it a fundamental component of the immune responses (Dash et al. 2018). LF can increase fish’s mucosal surface absorption of iron and other nutrients by stimulating mucosal secretions (Teraguchi et al. 2004; Embleton et al. 2013). Those authors have found that LF anti-microbial activity has been associated with its ability to efficiently regulate the iron uptake into the surface body or gut. This process seems to increase the body’s defense against invading infectious diseases caused by bacteria, viruses, and fungi (Embleton et al. 2013). The capability of LF to bind to important components of the G-ve bacteria, such as (lipopolysaccharides (LPS), porins, and outer membrane proteins) or the cell wall of G + ve bacteria may explain its antibacterial properties (Trybek et al. 2016). Furthermore, when an infection occurs, neutrophils store apo-LF inside the secondary granules to modulate the synthesis of pro-inflammatory cytokines (Drago-Serrano et al. 2017).

Certain peptides, like lactoferrampin and lactoferricin have a powerful defensive action. They have anti-microbial effects because of their hydrophobicity and cationic charge, making them important amphipathic molecules (Bellamy et al. 1992). Lactoferricin shows more powerful anti-fungal and antibacterial (Vorland et al. 1998), anti-microbial (Flores-Villaseñor et al. 2010; Drago-Serrano et al. 2017), anticancer (Gifford et al. 2005), and anti-inflammatory activities (Yan et al. 2013) than the intact BLF, while lactoferrampin displays a varied anti-microbial property against several parasites, bacteria, yeasts, and viruses (Gifford et al. 2005; Yan et al. 2013).

## *In -vitro* antibacterial properties of BLF

The antibacterial actions of BLF have been documented against many pathogens (Actor et al. 2009). The anti-microbial activity of BLF may be resulted from either (a) disruption of the cell wall of the bacterial cells or (b) enhancing bactericidal effects by the process of phagocytosis, which owe its ability to augment the synthesis of peroxidase enzyme (Drago-Serrano et al. 2017). Moreover, it was explained that the ability of BLF to increase bacterial killing in fish might be related to the substantially higher numbers of infiltrating neutrophils in both the spleen and liver. Neutrophils interrelating with liver tissues, such as Kupffer cells, can play a critical part in removing bacteria. Both lactoferrampin and lactoferricin have potent bactericidal activity (Bolscher et al. 2009). LF reveals bacteriostatic and bactericidal activities against a diversity of microbes (Niaz et al. 2019). BLF can bind with iron, depriving it that is needed for the growth of several bacterial pathogens such as *Bacillus stearothermophilus*, *Listeria monocytogenes*, *Escherichia coli*, *Bacillus subtilis*, *Salmonella* species, and *Shigella dysenteriae*, representing natural and effective anti-microbial mediator (Niaz et al. 2019).

## Benefits and applications of bovine lactoferrin (BLF) in aquaculture

Table 1 summarizes the biological effects of dietary BLF on the performances of several finfish and shrimp species in line with the published information.

**Table 1:** The biological effects of dietary bovine lactoferrin (BLF) on the performances of several finfish and shrimp species according to the published literature

Parameters	Fish species	Tested doses (mg/kg diet)	Feed duration	Effects	References
**1. Growth performance and feed efficiency**	Nile tilapia (*Oreochromis niloticus*)	200, 400 and 600	16 Wks	Improved growth indices, FER, FCR, and PEI	(Badawy and Al-Kenawy 2013)
	Asian Sea Bass (*Lates calcarifer*)	400, 800	8 Wks	Enhanced growth rate and FER	(Morshedi et al. 2021)
	Silvery-Black Porgy (*Sparidentex hasta*)	800 or 1200	8 Wks	Improved the growth indices and FER	(Pagheh et al. 2018)
	Siberian sturgeon (*Acipenser baeri*)	100, 200, 400, 800 and 1600	10 Wks	No effects on the fish growth	(Falahatkar et al. 2014)
**2. Haemato-biochemical indices**	Asian Sea Bass	400, 800	8 Wks	**↑** ALB and **↓** GLU levels in 800 mg/kg diet	(Morshedi et al. 2021)
	Yellowfin seabream (*Acanthopagrus latus*)	400, 800 and 1200	8 Wks	**↑** TP and ALB levels in fish fed higher level of BLF	(Esmaeili et al. 2019)
	Nile tilapia	800 and 1200	45 d	Enhanced ALP, ALT, and AST values Improved the RBCs and WBCs counts No effects on TP, ALB, GLO, BUN and creatinine	(Abdel-Wahab et al. 2021)
	Siberian sturgeon	100, 200, 400, 800 and 1600	10 Wks	A significantly positive impacts on stress response of fish **↓** Lactate and CORT levels	(Falahatkar et al. 2014)
	Amberjack (*Seriola dumerili*)	500, 1000, and 2000	4 Wks	Superior gill Na^+^/K^+^-ATPase activities Inferior plasma CORT amounts	(Yokoyama et al. 2019)
	Silvery-Black Porgy	800 or 1200	8 Wks	No effects on the haemato-immunological variables	(Pagheh et al. 2018)
	Siberian sturgeon	100, 200, 400, 800 and 1600	8 Wks	No effects on serum TP, GLO and ALB	(Eslamloo et al. 2012)
	African cichlid fish (*Sciaenochromis fryeri*)	100, 200, 400, 800 and 1600	8 Wks	No effects on serum TP, GLO and ALB	(Moradian et al. 2018)
	Japanese eel (*Anguilla japonica*)	500	3 Wks	No effects on serum TP	(Ren et al. 2007)
	Silver carp (*Hypophthalmichthys molitrix*)	600	30 d	**↑** TP, GLO, and ALB	(Soliman et al. 2022)
**3. Survivability after bacterial challenge**	Asian catfish	50, 100 and 200	2 Wks	Improved RPS after challenge with *A. hydrophila* than control	(Kumari et al. 2003)
	Nile tilapia	800 and 1200	45 d	Higher RPS after *A. hydrophila* challenge than control group	(Abdel-Wahab et al. 2021)
	Nile tilapia	800 and 1200	30 d	Higher RPS after *A. veronii* challenge than control	(Hashem et al. 2022)
	Yellowfin seabream	400, 800 and 1200	8 Wks	Higher RPS after *Vibrio harveyi* challenge than control	(Esmaeili et al. 2019)
	Rainbow trout (*Oncorhynchus mykiss*)	1000 and 10,000	35 d and 51 d	Higher RPS after *A. salmonicida achromogenes* than control	(Khuyen et al. 2017)
	Channel catfish (*Ictalurus punctatus*)	200, 400, 800, or 1600	5 Wks	Higher RPS after *Edwardsiella ictaluri* challenge than control	(Welker et al. 2010)
	Nile tilapia	200, 400, 800, or 1600	8 Wks	Higher RPS after *Streptococcus iniae* challenge than control	(Welker et al. 2007)
	Hybrid tilapia (*O. nilotica* × *O. mossambicus*)	10, 50, 100, and 150	60 d	Higher RPS after *Streptococcus agalactiae* challenge than control	(Wang et al. 2013)
	*Macrobrachium rosenbergii*	50, 100, and 200	7 or 14 d	Higher RPS after *A. hydrophila* challenge than control group	(Chand et al. 2006)
	Silver carp	600	30 d	Higher RPS after *Vibrio vulnificus* challenge than control	(Soliman et al. 2022)
**4. Immunity status**	Nile tilapia	200, 400 and 600	16 Wks	**↑** Serum LYZ and WBCs count	(Badawy and Al-Kenawy 2013)
	Nile tilapia	800 or 1200	30 d	**↑** expression of immune-related genes in spleen and kidney	(Hashem et al. 2022)
	Nile tilapia	800 and 1200	45 d	**↑** serum LYZ activity	(Abdel-Wahab et al. 2021)
	Siberian sturgeon	100, 200, 400, 800 and 1600	8 Wks	**↑** Serum bactericidal activity No effects on serum peroxidase, natural hemolytic complement, and IgM concentrations	(Eslamloo et al. 2012)
	Asian catfish	50, 100 and 200	2 Wks	**↑** serum LYZ activity	(Kumari et al. 2003)
	Nile tilapia	200, 400 and 600	4 Wks	**↑** serum LYZ activity	(El-Ashram and El-Boshy 2008)
	Japanese eel	500	3 Wks	**↑** serum and mucous LYZ activity	(Ren et al. 2007)
	Rainbow trout	50, 100, 200 and 400	8 Wks	**↑** serum LYZ activity	(Rahimnejad et al. 2012)
	Silver carp	600	30 d	**↑** Lymphocytes, and monocytes % **↑** PA and PI **↑** immune cells as lymphocytes in intestine **↑** immune cells as macrophages in liver, pancreas, and spleen	(Soliman et al. 2022)
	*Macrobrachium rosenbergii*	50, 100, and 200	7 or 14 d	100 mg/kg diet for 7 days showed significant increase in TP levels, agglutination titers against *A. hydrophila*, and PO activity Increased bacterial clearance 50 mg/kg diet for 7 or 14 days enhance PO activity	(Chand et al. 2006)
**5. Digestive enzymes and intestinal epithelial health**	Sobaity (*Sparidentex hasta*)	400 and 800	42 d	No effects on protease, amylase, and lipase No effects on the intestine bacterial flora	(Morshedi et al. 2016)
		400 or 1000	8 Wks	Reinforced and sustained the integrity of intestinal mucosa **↑** Total protease and amylase activities	(Morshedi et al. 2020)
**6. Iron absorption**	Siberian sturgeon	100, 200, 400, 800 and 1600	8 Wks	Iron absorption of fish influenced **↓** Plasma iron in all groups	(Eslamloo et al. 2012)
	Siberian sturgeon	100, 200, 400, 800 and 1600	10 Wks	High levels of BLFs decreased iron absorption	(Falahatkar et al. 2014)
**7. Antioxidants effects**	Nile tilapia	800 and 1200	45 d	Significantly **↑** SOD, CAT, and GSH	(Abdel-Wahab et al. 2021)
	Yellowfin seabream	400, 800 and 1200	8 Wks	No effects on CAT, GR, and GST enzymes	(Esmaeili et al. 2019)
	Asian Sea Bass	400, 800	8 Wks	800 mg kg reduction in the liver CAT activity 400 mg/kg diet improved the liver CAT activity	(Morshedi et al. 2021)
	Nile tilapia	800 and 1200	30 d	**↓** MDA concentrations **↑** serum TAOC after bacterial infection	(Hashem et al. 2022)
	Silvery-Black Porgy	800 or 1200	8 Wks	No effects on SOD, CAT, and TAOC in liver	(Pagheh et al. 2018)
**8. Expression of cytokines**	Rainbow trout	1000 and 10,000	35 d and 51 d	**↑** expression of *IL-1β* gene in BLF 0.1% group	(Khuyen et al. 2017)
	Nile tilapia	800 and 1200	45 d	Suppressed expression of *IFN-γ* gene **↑** expression of *IL-1β* gene	(Abdel-Wahab et al. 2021)
	Nile tilapia	800 or 1200	30 d	**↓** expression of *TLR9*, *TNF-α*, *IL-21*, *IL-6*, *IL-10*, *IFN-γ*, *IL-1β* and *caspase 3* genes	(Hashem et al. 2022)

**Abbreviations:** ALB: Albumin; ALP: Alkaline phosphatase; ALT: Alanine transaminase; AST: Aspartate transaminase; BUN: Blood urea nitrogen; CAT: Catalase; CORT: Cortisol; FCR: Feed conversion ratio; FER: Feed efficiency ratio; GLO: Globulin; GLU: Glucose; GSH: Reduced glutathione; GR: Glutathione reductase; GST: Glutathione S-transferase; IgM: Immunoglobulin M; IFN-γ: Interferon gamma; IL-10: Interleukin 10; *IL-1β*: Interleukin 1 beta; *IL-21*: Interleukin 21; *IL-6*: Interleukin 6; LYZ: Lysozyme activity; MDA: Malondialdehyde; PA: Phagocytic activity; PEI: protein efficiency index; PO: Phenoloxidase enzyme; PI: Phagocytic index; RBCs: Red blood cells; RPS: Relative percent survival; SOD: Superoxide dismutase; TAOC: Total antioxidant capacity; *TLR9*: Toll receptor 9; *TNF-α*: Tumor necrosis factor alpha; TP: Total protein; WBCs: White blood cells

### Impacts of BLF on iron metabolism in fish

Because of its vital function in oxygen transport and cellular respiration, iron is required by all higher vertebrates and also for fish (Eslamloo et al. 2012). It is widely known that the liver-derived peptide hepcidin regulates dietary iron absorption and iron transport from tissues into plasma (Raghuveer et al. 2002). LF has been shown to have a 300-fold greater affinity for iron than serum transferrin and its potential capacity to store iron across a wider pH range. It can also influence iron homeostasis by boosting iron export from the gastric tract and improving iron storage in ferritin (de Vet and Van Gool 1974). In fish, it was found that the iron absorption of Siberian sturgeon was substantially influenced as a response to dietary inclusion of BLF; thus, plasma iron concentrations in all BLF-treated groups significantly declined compared to the controls (Eslamloo et al. 2012). In the same context, the impacts of BLF on the iron absorption of Siberian sturgeon were decreased by increasing the dietary level of BLF to more than 0.8 g/kg (Falahatkar et al. 2014). Moreover, the iron-binding capability was augmented in fish fed with a 0.8 g BLF/kg diet (Eslamloo et al. 2012). It seems that the capacity of BLF to enhance iron absorption depends on an organism’s physiological condition, aquatic conditions, environmental impacts, and the levels of iron in the diet. The reports established on the effect of BLF on iron absorption in fish are restricted, and the consequences of the investigations on mammals are also varying.

### Impacts of BLF on growth

Reports showed that the dietary application of BLF enhanced the growth, feed efficiency ratio, feed conversion, and protein efficiency index in Nile tilapia (Badawy and Al-Kenawy 2013). Fish species differences may contribute to the differences in the results of BLF on fish growth. Kakuta (1996) also indicated that dietary supplementation of BLF at a level 1 g/kg diet significantly improved the growth of goldfish (*Carassius auratus*). Similarly, it was found that Asian Sea Bass fed diets supplied with BLF 0.8 g/kg diet showed enhanced growth indices via improving feed efficiency and growth rate (Morshedi et al. 2021). Furthermore, Pagheh et al. (2018) demonstrated that Silvery-Black Porgy fed with 0.8 g of BLF per kg diet had greater improvement in the growth indices and feed efficiency compared with 1.2 g of BLF /kg diet and control groups. In several other fish species, it was also illustrated that dietary BLF could increase the growth rates in several finfish species, as in Atlantic salmon (Lygren et al. 1999), common carp (Kakuta 1998), Japanese flounder (Yokoyama et al. 2005), orange-spotted grouper (Yokoyama et al. 2006), Siberian sturgeon (Eslamloo et al. 2012), and Nile tilapia (Abdel-Wahab et al. 2021). Even though several studies have shown that dietary BLF positively impacts the growth indices in several fish species, Falahatkar et al. (2014) declared that dietary BLF did not influence the growth performance of Siberian sturgeon (*Acipenser baeri*).

In the same context, reports suggested that the relationship between BLF and some other molecules in the feed, like iron, might influence BLF absorption and enhance its biological functions (Yokoyama et al. 2005). Also, the benefits of BLF on growth indices might be linked with the ability of BLF the stimulation of digestive enzyme secretions. A third hypothesis presented that dietary BLF improved the proliferation of enterocytes and safeguarded intestinal villous structure and crypt (Li et al. 2014; Nguyen et al. 2013). Nonetheless, the hypotheses mentioned above, the precise mechanisms of improvement of fish growth performance by dietary BLF, are still unclear.

### Effects of BLF on the digestive enzymes and intestinal epithelial health

Little information was reported on the effects of BLF on the intestinal health, microbiota, and histomorphometry of treated fish. A previously published study performed by Morshedi et al. (2016) presented that dietary BLF neither affects the digestive enzyme activities (protease, amylase, and lipase) nor affects the intestinal flora in Sobaity (*Sparidentex hasta*). However, in the same fish species, it was found that combined treatment with BLF and *Lactobacillus plantarum* reinforced and sustained the integrity of intestinal mucosa, resulted in intestinal brush border equilibrium, and increased the levels of total protease and amylase activities in the gut cavity by interrelating with LF receptors (Morshedi et al. 2020). Thus, these points warrant additional investigations.

### Effects of BLF on haemato-biochemical indices

Hematological and serum biochemical markers are critical clinical tools for diagnosing the overall health state of fish (Fazio 2019; Naiel et al. 2021a). Several studies have reported considerable impacts of BLF on some biochemical blood indices of fish, such as blood proteins, serum metabolites, blood indices, and stress biomarkers. For example, it was found that adding BLF (800 mg/kg) to the Asian sea bass diets produced significantly high levels of serum albumin (ALB) and lower glucose (GLU) levels compared with those in control and 400 mg/kg diet (Morshedi et al. 2021). Moreover, it was declared that dietary BLF alone or combined with nano-chitosan significantly augmented liver function through enhanced ALP, ALT, and AST enzyme activities in comparison with the free-BLF group (Abdel-Wahab et al. 2021). Compared with the control one it was found that fish received diets supplemented with BLF revealed significantly superior gill Na^+^/K^+^-ATPase activities and low plasma cortisol (CORT) amounts (Yokoyama et al. 2019). However, another study showed that adding 800 or 1200 mg of BLF /kg diet did not induce any significant alterations in the haemato-immunological variables of Silvery-Black Porgy fish (Pagheh et al. 2018). Laterally, Hashem et al. (2022) explained that the dietary BLF (800 mg/kg) significantly increased RBCs and total WBCs counts of tilapia fish. These enhancements in hematological parameters may be ascribed to the dietary roles of BLF. BLF, as an iron-binding glycoprotein, can restore iron levels in diets which may consequently enhance the fish’s health status. In human medical research, it was previously reported that dietary LF could treat iron-deficiency anemia in human beings (Morton 2019) and enhance the iron status of infants and pregnant women (Lönnerdal 2009). BLF also fortified iron metabolic homeostasis and positively impacted infants’ hemoglobin and iron status (Ke et al. 2015).

BLF effects on the fish’ blood protein fractions are controversial. Esmaeili et al. (2019) presented that total protein (TP) and ALB levels were increased in yellowfin seabream that fed a diet supplied with a higher level of BLF (1200 mg/kg diet). Newly published research conducted by Soliman et al. (2022) presented that dietary BLF (600 mg/kg diet for 30 days) considerably increased total protein (TP), globulin (GLO), and ALB levels in silver carp (*Hypophthalmichthys molitrix*). Differently, another research study revealed that the inclusion of a higher level of BLF in the diets of Nile tilapia did not noticeably influence serum biochemical indices like TP, ALB, and GLO concentrations (Abdel-Wahab et al. 2021). In the same way, Eslamloo et al. (2012) stated that the different levels of dietary BLF did not exhibit any significant changes in serum protein fraction (TP, ALB, and GLO) of Siberian sturgeon. In an earlier study, it was observed that no alterations were found in the serum TP concentrations of Japanese eels that received diets incorporated with BLF alone or combined with vitamin C (Ren et al. 2007; Moradian et al. 2018) noted that there were no substantial influences of various levels of dietary BLF on blood protein fractions of African cichlid fish. These inconsistencies might be owing to several factors, such as fish species alterations, dosage effects, experimental systems, *etc*. Thus, further extra studies, such as molecular studies, are necessary to elucidate the factors that led to these differences.

From another point of view, several reports proved the ability of BLF-enriched diets to alleviate the stress markers in several fish species (Luna-Castro et al. 2022). BLF positively influenced blood GLU and CORT levels in carp (*Cyprinus carpio*) (Kakuta 1998). Moreover, the supplementation of BLF within common carp and Japanese flounder diets at level 0.6 g per kg can moderate the plasma CORT levels within desirable borders for supportive stress resistance (Hashem et al. 2022; Kakuta 1998; Yokoyama et al. 2005). Interestingly, a significantly positive impact of BLF was detected in the stress response, such as lactate and CORT levels of Siberian sturgeon (Falahatkar et al. 2014). From the findings mentioned above, we can conclude that dietary BLF could enhance stress tolerance, hematological profile, liver functions and renal functions of treated fish with possible applicability in fish diets.

### Antioxidant properties of BLF

The enzymatic antioxidant defensive mechanisms are important in counteracting the oxidative stress that occurs from the overproduction of free radicals and reactive oxygen species (ROS). Studies showed that dietary LF administration was related to the increased antioxidant capacity of healthy fish (Lygren et al. 1999). Dietary administration of both BLF and chitosan nanoparticles significantly improved the superoxide dismutase (SOD), catalase (CAT), and glutathione S-transferase (GST) enzyme levels in Nile tilapia (Abdel-Wahab et al. 2021). However, dietary BLF supplementation did not affect the CAT, GST, and glutathione reductase (GSR) activities of yellowfin sea bream (Esmaeili et al. 2019). While Morshedi et al. (2021) suggested that the high dose of BLF (800 mg/kg diet) significantly reduced liver CAT activity, while a 400 mg/kg diet improved the CAT activity in the liver of Asian sea bass. Hashem et al. (2022) recently demonstrated that Nile tilapia diets supplied with 800 mg BLF/kg diet significantly reduced the serum MDA and significantly increased serum total antioxidant capacity (TAOC) after bacterial infection. In contrast, Pagheh et al. (2018) illustrated that the dietary addition of BLF (800 or 1200 mg/kg diet) did not affect the liver antioxidant indices, including SOD, CAT, and TAOC of Silvery-Black Porgy.

The antioxidative capability of fish that received BFL in their diets could be accredited by the chelating and scavenging properties of BLF against oxidative stress. Reports showed that the antioxidant properties of BLF have been linked to the prevention of lipid peroxidation and erythrocyte hemolysis (Morshedi et al. 2021). Besides, LF administration resulted in lower intracellular levels of ROS, indicating its capacity to prevent oxidative stress (Hashem et al. 2022). Moreover, LF has metal ion binding ability and may prevent iron-catalyzed hydroxyl radicals via the Fenton reaction, which is considered a major source of ROS. Hence, the LF antioxidant function is most likely connected to its capacity to scavenge iron and reduce ROS production (Esmaeili et al. 2019).

### Effects of BLF on the expression of cytokines

Cytokines are signaling molecules formed by immune cells that increase the influx of phagocytic cells to overcome and destroy attacking pathogens. They display a significant function in regulating the fish’s immune response. Interleukin 1 beta (*IL-1β*), as a pro-inflammatory cytokine, reveals a substantial part in regulating inflammatory and immune processes through a contribution to the encouragement of the proliferation of macrophages and lymphocytes (Wang and Secombes 2013). Reports showed that BLF could decrease the inflammatory process in various pathologies. It has been known that BLF could suppress different inflammatory agents, such as TNF and CD4 cells. Specifically, LF might attach and sequester lipopolysaccharides, avoiding pro-inflammatory pathway activation, sepsis, and tissue damage (Siqueiros-Cendón et al. 2014).

In aquaculture, several studies have been published on the impacts of dietary BLF on the expression of cytokines. For instance, the dietary application of 0.1% BLF augmented the expression of the *IL-1β* gene in the kidney of rainbow trout juveniles (Khuyen et al. 2017). Also, supplementing diets with BLF alone or with a mixture with nano-chitosan suppressed the expression of tumor necrosis factor-alpha (*TNF-α*) and up-regulated expression of *IL-1β* genes in Nile tilapia (Abdel-Wahab et al. 2021). Newly published research in Nile tilapia proved that BLF-supplemented diets produced downregulation in mRNA expression levels of toll-like receptor 9 (*TLR9*), *TNF-α*, *IL-21*, *IL-6*, *IL-10*, *IFN-γ*, *IL-1β*, and *caspase3* in comparison with those reared in the oxytetracycline treated group (Hashem et al. 2022). Those authors suggested the downregulation trend of these inflammatory indicators in tilapia fed diet supplied with 1.2 g BLF / kg diet compared to the control group (Hashem et al. 2022).

### Immune-stimulant effects of BLF

In the era of green-friendly industry, using natural immune stimulants in the aquaculture sector to avoid bacterial diseases is regarded as a new positive approach (Kumari et al. 2003; El-Saadony et al. 2021; Naiel et al. 2021b; Yilmaz et al. 2022). Research has shown that BLF is one of the attractive elements in bovine milk, which has potent immunostimulatory effects (Niaz et al. 2019). LF that has less than 5% iron saturation is termed as “apo-lactoferrin” (apo-LF or the native iron free), while the iron-saturated lactoferrin is termed as “holo-lactoferrin’ (holo-LF) (Bokkhim et al. 2013). LF exhibits potent immune modulating functions in mammals (Suzuki et al. 2005). BLF can secrete more anti-inflammatory cytokines and robust pro-inflammatory responses in the animal gut (Donovan 2016). While in fish, the immune stimulatory activity of BLF is facilitated by triggering non-specific immunity, which offers defense in the face of a wide variety of fish-associated pathogens (Cecchini and Caputo 2009).

It is well-recognized that the transcription of immune-associated genes can be a beneficial tool for assessing immune responses in aquatic animals (Alhoshy et al. 2022). The up-regulated expression of immune-associated genes in fish groups that fed BLF-supplied diets may be linked with its ability to stimulate the production of cytokines through macrophages and also increase the production of macrophages, granulocytes, and neutrophils (Sakai et al. 1993). Dietary addition of BLF stimulated more noticeably the transcript of immune-linked genes. The increased expression of the immune-associated genes could elucidate the increased resistance in rainbow trout juveniles that were previously fed BLF-based diets (Khuyen et al. 2017).

It was also noticed that BLF-based diets significantly increased mucus secretion and serum bactericidal activities in Siberian sturgeons. However, other serum peroxidases, natural hemolytic complement, and total IgM concentrations were not affected by dietary BLF supplementation (Eslamloo et al. 2012). Previously published research studies demonstrated that dietary BLF boosted the lysosome activity in a range of finfish species such as Asian catfish (Kumari et al. 2003), Nile tilapia (El-Ashram and El-Boshy 2008), Japanese eel (Ren et al. 2007), rainbow trout (Rahimnejad et al. 2012), Siberian sturgeon (Eslamloo et al. 2012), African cichlid fish (Moradian et al. 2018), Silvery-black Porgy (Pagheh et al. 2018), and yellowfin sea bream (Esmaeili et al. 2019) and Asian sea bass (Morshedi et al. 2021; Yokoyama et al. 2019) found that fish fed with 1 of 2 g/kg diet of BLF exhibited a superior level of mucus LYZ activity than the control group. Recently, Abdel-Wahab et al. (2021) described that serum LYZ activity as augmented in Nile tilapia fed with BLF,while the highest levels were noticed in the fish group that fed a combination of BFL and chitosan nanoparticles.

In tilapia fish, the immunological variables such as IgM and IgG were significantly augmented by dietary inclusion of 0.8 or 1.2 g /kg BLF (Hashem et al. 2022). Inversely, Welker et al. (2007) presented BLF-supplementation did not influence that serum LYZ levels in Nile tilapia diets. Also, the values of LYZ activity in seabream fed with BLF did not present substantial fluctuations compared with the prebiotic and control groups (Morshedi et al. 2020). These inconsistencies in the literature may be connected with factors such as BLF doses, water quality, experimental conditions, fish species, and pepsin activities in fish stomachs, which may affect their capability to digest BLF into the intestinal lumen, thus, affecting the biological availability of BLF.

A recently published paper by Soliman and coauthors showed that dietary BLF significantly increased cell-mediated immunity in silver carp (Soliman et al. 2022). Those authors found that dietary BLF significantly increased lymphocytes and monocytes %, phagocytic capacity (phagocytic index and phagocytic activity), and the number of lymphocytes in the intestine and macrophages in the liver, pancreas, and spleen of silver carp (Soliman et al. 2022). In shrimp, a formerly published study also showed that diets supplied with BLF at a dose rate of 100 mg/kg diet for seven days induced a significant increase in agglutination titers against *A. hydrophila* and phenoloxidase enzyme activity in *Macrobrachium rosenbergii* (Chand et al. 2006).

### Roles of BLF for enhancement of resistance against bacterial infections

It has been reviewed that BLF can boost the fish’ immune system and increase disease resistance after bacterial challenge (Luna-Castro et al. 2022). Reports showed that the dietary application of BLF can modify the immunity of the intestinal mucosa, therefore, may help to increase resistance against bacterial infections (Taherah 2021). In the same sense, the application of BLF into the diets of Asian catfish (*Clarias batrachus*) significantly improved the survivability after the challenge with *A. hydrophila* bacteria compared to the non-BLF-supplemented fish (Kumari et al. 2003). Similarly, it was reported that BLF boosted the resistance against bacterial infections in several fish species, such as *Edwardsiella ictaluri* in channel catfish (Welker et al. 2010), *Streptococcus agalactiae* in hybrid tilapia (*O. nilotica* × *O. mossambicus*) (Wang et al. 2013), A. *salmonicida achromogenes* in rainbow trout (Khuyen et al. 2017), *V.harveyi* in yellowfin sea bream (Esmaeili et al. 2019), and recently *V. vulnificus* in silver carp (*Hypophthalmichthys molitrix*) (Soliman et al. 2022). Also, in shrimp species, it was found that dietary LF significantly enhanced the disease resistance of *Macrobrachium rosenbergii* and the survival rates after *A. hydrophila* challenge (Chand et al. 2006).

An earlier report showed that the *in vivo* antibacterial properties of BLF could be ascribed to the antimicrobic activity of BLF via the promotion of iron essential for bacterial growth, which will then lead to the suppression of bacterial growth (González-Chávez et al. 2009). In the same sense, it was found that dietary BLF boosted the resistance against bacterial infections of Nile tilapia fish such as *Streptococcus iniae* (Welker et al. 2007) and, recently, *A. veronii* (Hashem et al. 2022). Lately, it was also found that Nile tilapia that received diets enriched with graded amounts of BLF alone or combined with chitosan nanoparticles had considerably higher relative percentage survival values after experimental infection with *A. hydrophila* when than the control group (Abdel-Wahab et al. 2021).

### Anti-parasitic properties of BLF

**Fig. 2:**
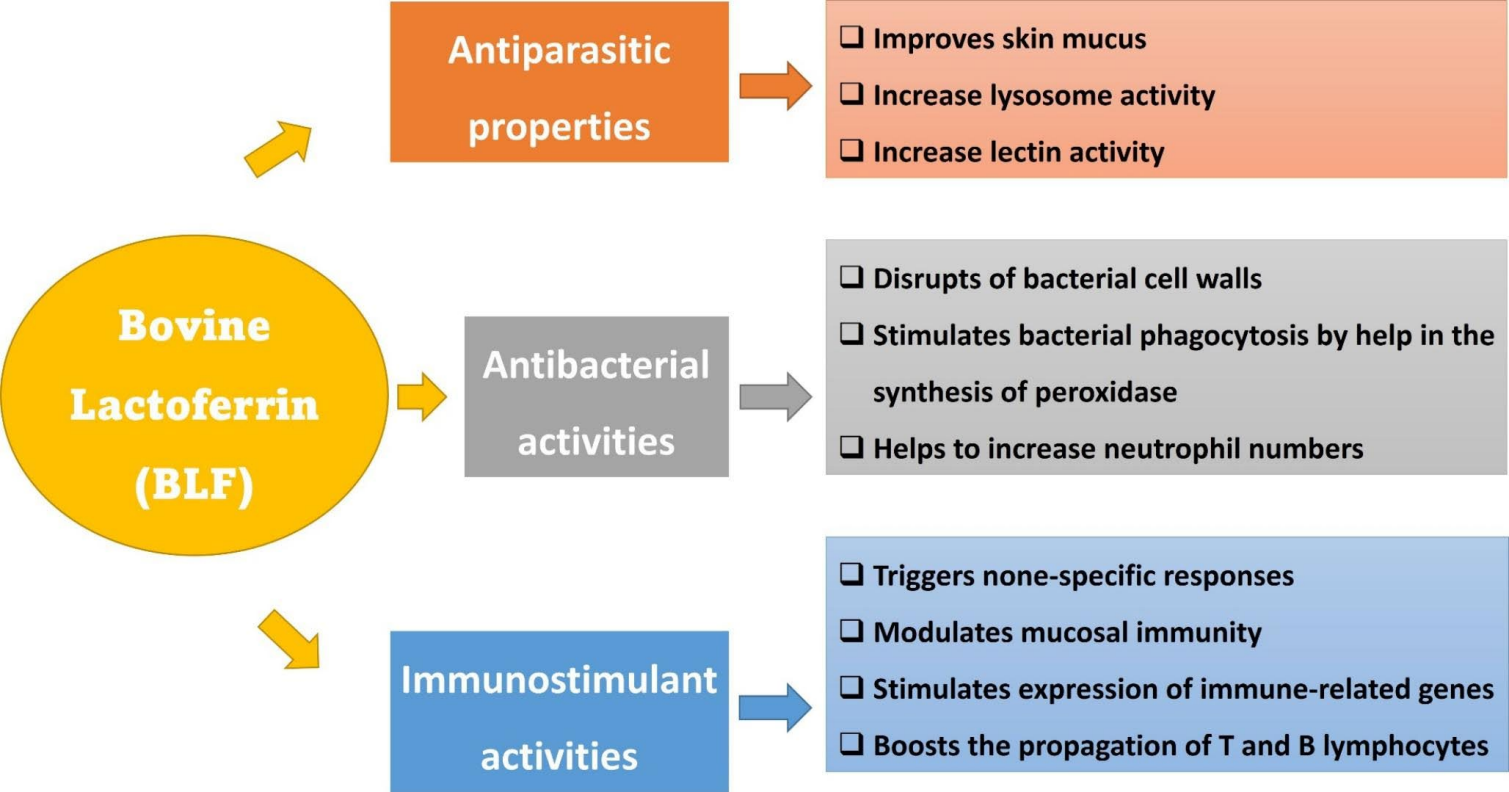
The antibacterial, antiparasitic and immunostimulant properties of dietary bovine lactoferrin (BLF) in fish

In the studies conducted in human medicine, it was suggested that the anti-parasitic properties of BLF seem to be linked with the interference in the iron hemostasis of *Pneumocystis carinii* (Cirioni et al. 2000), or sometimes, BLF is represented as a specific iron donor in other parasites such as *Tritrichomonas foetus* (Giansanti et al. 2013). Studies conducted in the *in vitro* trials revealed that LF has a verifiable activity concerning human pathogenic fungi, like different *Candida* species, and could suppress the growth of *Plasmodium berghei* (Larkins 2005). Figure 2 represents the proposed the anti-parasitic activities of BLF.

In fish, it has been proposed that dietary inclusion of BLF has positive impacts against different ectoparasites such as *Ichthyophthirius multifiliis* and *Cryptocaryon irritans* that infects the fish body surfaces (Kakuta 1996, 1998). It also improves skin mucus secretion, LYZ activity, and lectin activities in red sea bream (Kakuta 1996) and orange-spotted grouper (*Epinephelus coioides*) (Yokoyama et al. 2006). Lately, it was observed that *Neobenedenia girellae* fed a diet enriched with 1 g/kg BLF had fewer parasites than those provided in the control diet (Yokoyama et al. 2019). In addition, those authors also found that the number of parasites per unit area of the fish body surface was inferior in the fish that received dietary BLF than the control group. The dietary application of BLF enhanced the lectin activities in the dermal mucus and may remove the chance of recombinant pathogen binding to the fish body. As known, lectin is recognized as the main issue associated with the anti-parasitic activity of the fish body surface. It has also been indicated that the skin mucus lysozyme activity was noticeably augmented in fish that received BLF. This enhancement of lysozyme activity was connected with a low rate of *N. girellae* infection (Yokoyama et al. 2019).

## Conclusions and perspective

This article described the potential uses of BLF as a bio-feed additive in aquaculture. It also highlighted the prominent effects of dietary BLF on the growth indices, digestive enzymes, feed efficiency, iron metabolism, blood metabolites, immunity, disease resistance, antioxidant status, and expression of pro-inflammatory responses in treated fish and shrimp. Based on the literature cited, BLF may be used as an alternative to an antibiotic application. In addition, it can be used as a feed supplement for reducing the negative impacts of stressful conditions affecting fish and shrimp. These functions may be linked with its potential anti-microbial, anti-inflammatory, anti-parasitic, and antiviral activities. Although those mentioned above are vital biological activities of BLF, the actual mechanisms of BLF in improving fish health still require additional investigations and research studies.

## Data Availability

Not applicable.

## Data Availability

The authors confirm that the data supporting the findings of this study are available upon reasonable request from the corresponding author.

## References

[CR1] Abdel-Latif HMR, Dawood MAO, Alagawany M, Faggio C, Nowosad J, Kucharczyk D (2022). Health benefits and potential applications of fucoidan (FCD) extracted from brown seaweeds in aquaculture: an updated review. Fish Shellfish Immunol.

[CR2] Abdel-Latif HMR, Dawood MAO, Menanteau-Ledouble S, El-Matbouli M (2020). The nature and consequences of co-infections in tilapia: a review. J Fish Dis.

[CR3] Abdel-Latif HMR, El-Ashram S, Yilmaz S, Naiel MAE, Abdul Kari Z, Hamid NKA, Kucharczyk D (2022). The effectiveness of *Arthrospira platensis* and microalgae in relieving stressful conditions affecting finfish and shellfish species: an overview. Aquaculture Rep.

[CR4] Abdel-Tawwab M, Khalil RH, Nour AM, Elkhayat BK, Khalifa E, Abdel-Latif HMR (2022). Effects of *Bacillus subtilis*-fermented rice bran on water quality, performance, antioxidants/oxidants, and immunity biomarkers of white leg shrimp (*Litopenaeus vannamei*) reared at different salinities with zero water exchange. J Appl Aquac.

[CR5] Abdel-Wahab MM, Taha NM, Lebda MA, Elfeky MS, Abdel-Latif HMR (2021). Effects of bovine lactoferrin and chitosan nanoparticles on serum biochemical indices, antioxidative enzymes, transcriptomic responses, and resistance of Nile tilapia against *Aeromonas hydrophila*. Fish Shellfish Immunol.

[CR6] Actor JK, Hwang SA, Kruzel ML (2009). Lactoferrin as a natural immune modulator. Curr Pharm Des.

[CR7] Adlerova L, Bartoskova A, Faldyna M (2008). Lactoferrin: a review. Vet Med.

[CR8] Ahmadifar E, Yousefi M, Karimi M, Fadaei Raieni R, Dadar M, Yilmaz S, Abdel-Latif HMR (2021). Benefits of Dietary Polyphenols and Polyphenol-Rich additives to aquatic Animal Health: an overview. Reviews in Fisheries Science & Aquaculture.

[CR9] Alagawany M, Farag MR, Abdelnour SA, Dawood MAO, Elnesr SS, Dhama K (2021). Curcumin and its different forms: a review on fish nutrition. Aquaculture.

[CR10] Alhoshy M, Shehata AI, Habib YJ, Abdel-Latif HMR, Wang Y, Zhang Z (2022). Nutrigenomics in crustaceans: current status and future prospects. Fish Shellfish Immunol.

[CR11] Anderson DP (1992). Immunostimulants, adjuvants, and vaccine carriers in fish: applications to aquaculture. Annu Rev Fish Dis.

[CR12] Badawy TE-S, Al-Kenawy D (2013). Assessment of immune response supplemental immunoton and bovine lactoferrin as alternatives to antibiotics in Nile tilapia (*Oreochromis niloticus*). J Arab Aquaculture Soc.

[CR13] Baker EN, Baker HM (2009). A structural framework for understanding the multi-functional character of lactoferrin. Biochimie.

[CR14] Bellamy W, Takase M, Wakabayashi H, Kawase K, Tomita M (1992). Antibacterial spectrum of lactoferricin B, a potent bactericidal peptide derived from the N-terminal region of bovine lactoferrin. J Appl Bacteriol.

[CR15] Bokkhim H, Bansal N, GrØndahl L, Bhandari B (2013). Physico-chemical properties of different forms of bovine lactoferrin. Food Chem.

[CR16] Bolscher JGM, Adão R, Nazmi K, van den Keybus PAM, van ’t Hof W, Nieuw Amerongen AV, Veerman ECI (2009). Bactericidal activity of LFchimera is stronger and less sensitive to ionic strength than its constituent lactoferricin and lactoferrampin peptides. Biochimie.

[CR17] Buccigrossi V, de Marco G, Bruzzese E, Ombrato L, Bracale I, Polito G, Guarino A (2007). Lactoferrin induces concentration-dependent functional modulation of intestinal proliferation and differentiation. Pediatr Res.

[CR18] Cecchini S, Caputo AR (2009). Serum disposition of bovine lactoferrin after oral and anal administration and its proteolytic cleavage by gastric transit in rainbow trout (*Oncorhynchus mykiss* W.). Fish Shellfish Immunol.

[CR19] Chand RK, Sahoo PK, Kumari J, Pillai BR, Mishra BK (2006). Dietary administration of bovine lactoferrin influences the immune ability of the giant freshwater prawn *Macrobrachium rosenbergii* (de Man) and its resistance against *Aeromonas hydrophila* infection and nitrite stress. Fish Shellfish Immunol.

[CR20] Cirioni O, Giacometti A, Barchiesi F, Scalise G (2000). Inhibition of growth of *Pneumocystis carinii* by lactoferrins alone and in combination with pyrimethamine, clarithromycin and minocycline. J Antimicrob Chemother.

[CR21] Dash S, Das SK, Samal J, Thatoi HN (2018). Epidermal mucus, a major determinant in fish health: a review. Iran J Vet Res.

[CR22] De Vet BJCM, Van Gool J (1974). Lactoferrin and iron absorption in the small intestine. Acta Med Scand.

[CR23] Donovan SM (2016) The role of lactoferrin in gastrointestinal and immune development and function: a preclinical perspective. J Pediatr 173. 10.1016/j.jpeds.2016.02.072. S16-S28 10.1016/j.jpeds.2016.02.072 27234407

[CR24] Drago-Serrano ME, Campos-Rodríguez R, Carrero JC, De la Garza M (2017). Lactoferrin: Balancing Ups and Downs of inflammation due to Microbial Infections. Int J Mol Sci.

[CR25] El-Ashram A, El-Boshy M (2008) Assessment of dietary bovine Lactoferrin in enhancement of immune function and disease resistance in Nile tilapia (*Oreochromis niloticus*). Paper presented at the The Eighth International Symposium on Tilapia in Aquaculture

[CR26] El-Saadony MT, Alagawany M, Patra AK, Kar I, Tiwari R, Dawood MAO, Abdel-Latif HMR (2021). The functionality of probiotics in aquaculture: an overview. Fish Shellfish Immunol.

[CR27] Elzoghby AO, Abdelmoneem MA, Hassanin IA, Abd Elwakil MM, Elnaggar MA, Mokhtar S, Elkhodairy KA (2020). Lactoferrin, a multi-functional glycoprotein: active therapeutic, drug nanocarrier & targeting ligand. Biomaterials.

[CR28] Embleton ND, Berrington JE, McGuire W, Stewart CJ, Cummings SP (2013). Lactoferrin: anti-microbial activity and therapeutic potential. Seminars in Fetal and Neonatal Medicine.

[CR29] Eslamloo K, Falahatkar B, Yokoyama S (2012). Effects of dietary bovine lactoferrin on growth, physiological performance, iron metabolism and non-specific immune responses of siberian sturgeon *Acipenser baeri*. Fish Shellfish Immunol.

[CR30] Esmaeili A, Sotoudeh E, Morshedi V, Bagheri D, Dorafshan S (2019). Effects of dietary supplementation of bovine lactoferrin on antioxidant status, immune response and disease resistance of yellowfin sea bream (*Acanthopagrus latus*) against *Vibrio harveyi*. Fish Shellfish Immunol.

[CR31] Falahatkar B, Eslamloo K, Yokoyama S (2014). Suppression of stress responses in siberian sturgeon, *Acipenser baeri*, Juveniles by the Dietary Administration of bovine lactoferrin. J World Aquaculture Soc.

[CR32] Farag MR, Abdelnour SA, Patra AK, Dhama K, Dawood MAO, Elnesr SS, Alagawany M (2021). Propolis: Properties and composition, health benefits and applications in fish nutrition. Fish Shellfish Immunol.

[CR33] Fazio F (2019). Fish hematology analysis as an important tool of aquaculture: a review. Aquaculture.

[CR34] Fernandes KE, Carter DA (2017) The antifungal activity of Lactoferrin and its derived peptides: mechanisms of action and synergy with drugs against fungal pathogens. Front Microbiol 8. doi:10.3389/fmicb.2017.00002 10.3389/fmicb.2017.00002 PMC5241296 28149293

[CR35] Flores-Villaseñor H, Canizalez-Román A, Reyes-Lopez M, Nazmi K, de la Garza M, Zazueta-Beltrán J, Bolscher JM (2010). Bactericidal effect of bovine lactoferrin, LFcin, LFampin and LFchimera on antibiotic-resistant *Staphylococcus aureus* and *Escherichia coli*. Biometals.

[CR36] Founou LL, Founou RC, Essack SY (2016) Antibiotic resistance in the Food Chain: a developing country-perspective. Front Microbiol 7. doi:10.3389/fmicb.2016.01881 10.3389/fmicb.2016.01881 PMC5120092 27933044

[CR37] Franco I, Pérez MD, Conesa C, Calvo M, Sánchez L (2018). Effect of technological treatments on bovine lactoferrin: an overview. Food Res Int.

[CR38] García-Montoya IA, Cendón TS, Arévalo-Gallegos S, Rascón-Cruz Q (2012). Lactoferrin a multiple bioactive protein: an overview. Biochimica et Biophysica Acta (BBA). Gen Subj.

[CR39] Giansanti F, Leboffe L, D’Elia I, Antonini G (2013). An update on the anti-fungal activities of lactoferrin: new promising applications in diagnostic, therapeutics and biotechnology. Anti-Infective Agents.

[CR40] Gifford JL, Hunter HN, Vogel HJ (2005). Lactoferricin. Cell Mol Life Sci.

[CR41] González-Chávez SA, Arévalo-Gallegos S, Rascón-Cruz Q (2009) Lactoferrin: structure, function and applications. International Journal of Antimicrobial Agents 33(4):301.e301-301.e308. doi:10.1016/j.ijantimicag.2008.07.020 10.1016/j.ijantimicag.2008.07.020 18842395

[CR42] Hao Y, Yang N, Teng D, Wang X, Mao R, Wang J (2018). A review of the design and modification of lactoferricins and their derivatives. Biometals.

[CR43] Harada E, Itoh Y, Sitizyo K, Takeuchi T, Araki Y, Kitagawa H (1999). Characteristic transport of lactoferrin from the intestinal lumen into the bile via the blood in piglets. Comp Biochem Physiol A: Mol Integr Physiol.

[CR44] Hashem NMA, El-Son MAM, Ateya AI, Saleh RM (2022). Impact of lactoferrin supplementation on oxidative stress, gene expression and immunity dysfunction induced by *Aeromonas veronii* in Nile tilapia (*Oreochromis niloticus*). Aquac Res.

[CR45] Kakuta I (1996). Protective effect of orally administrated bovine lactoferrin against experimental infection of goldfish *Carassius auratus* with *Ichthyophthirius multifiliis*. Aquaculture Sci.

[CR46] Kakuta I (1998) Reduction of stress response in carp, *Cyprinus carpio* L., held under deteriorating environmental conditions, by oral administration of bovine lactoferrin. Journal of Fish Diseases 21(3): 161–167. doi:10.1046/j.1365-2761.1998.00087.x 10.1046/j.1365-2761.1998.00087.x 21361970

[CR47] Ke C, Lan Z, Hua L, Ying Z, Humina X, Jia S, Meng M (2015). Iron metabolism in infants: influence of bovine lactoferrin from iron-fortified formula. Nutrition.

[CR48] Khuyen TD, Mandiki SNM, Cornet V, Douxfils J, Betoulle S, Bossier P, Kestemont P (2017). Physiological and immune response of juvenile rainbow trout to dietary bovine lactoferrin. Fish Shellfish Immunol.

[CR49] Krewulak KD, Vogel HJ (2008). Structural biology of bacterial iron uptake. Biochimica et Biophysica Acta (BBA). - Biomembr.

[CR50] Kumari J, Swain T, Sahoo PK (2003). Dietary bovine lactoferrin induces changes in immunity level and disease resistance in asian catfish *Clarias batrachus*. Vet Immunol Immunopathol.

[CR51] Morton A (2019). Lactoferrin and iron deficiency anaemia in pregnancy. Australian J Gen Pract.

[CR52] Lall SP, Kaushik SJ (2021) Nutrition and Metabolism of Minerals in Fish. Animals11(9): 2711. 10.3390/ani11092711 10.3390/ani11092711 PMC8466162 34573676

[CR53] Larkins N (2005). Potential implications of lactoferrin as a therapeutic agent. Am J Vet Res.

[CR54] Li Q, Hu W, Zhao J, Wang J, Dai Y, Zhao Y, Li N (2014). Supplementation transgenic cow’s milk containing recombinant human lactoferrin enhances systematic and intestinal immune responses in piglets. Mol Biol Rep.

[CR55] Liu W, Ye A, Liu W, Liu C, Singh H (2013). Stability during in vitro digestion of lactoferrin-loaded liposomes prepared from milk fat globule membrane-derived phospholipids. J Dairy Sci.

[CR56] Lönnerdal B (2009). Nutritional roles of lactoferrin. Current opinion in Clinical Nutrition &. Metabolic Care.

[CR57] Lönnerdal B, Du X, Jiang R (2020). Biological activities of commercial bovine lactoferrin sources. Biochem Cell Biol.

[CR58] Luna-Castro S, Ceballos-Olvera I, Benavides-González F, Blanco-Martínez Z, Sánchez-Martínez G, de la Garza M (2022) Bovine lactoferrin in fish culture: Current research and future directions. Aquaculture Research 53(3):735–745. doi:10.1111/are.15621

[CR59] Lygren B, Sveier H, Hjeltnes B, Waagbø R (1999). Examination of the immunomodulatory properties and the effect on disease resistance of dietary bovine lactoferrin and vitamin C fed to Atlantic salmon (*Salmo salar*) for a short-term period. Fish Shellfish Immunol.

[CR60] Manyi-Loh C, Mamphweli S, Meyer E, Okoh A (2018). Antibiotic use in Agriculture and its consequential resistance in environmental sources: potential Public Health Implications. Molecules.

[CR61] Moradian AM, Dorafshan S, Paykan Heyrati F, Ebrahimi E (2018). Effects of dietary bovine lactoferrin on growth, haemato-biochemical parameters, immune functions and tolerance to air exposure stress in the african cichlid *Sciaenochromis fryeri*. Aquacult Nutr.

[CR62] Moreno-Expósito L, Illescas-Montes R, Melguizo-Rodríguez L, Ruiz C, Ramos-Torrecillas J, de Luna-Bertos E (2018). Multi-functional capacity and therapeutic potential of lactoferrin. Life Sci.

[CR63] Morshedi V, Agh N, Marammazi J, Noori F, Mohamadian T (2016). Effects of different levels of dietary lactoferrin on digestive enzymes, body composition and intestine bacterial flora of sobaity (*Sparidentex hasta*) fingerling. Veterinary Researches & Biological Products.

[CR64] Morshedi V, Agh N, Noori F, Jafari F, Ghasemi A, Mozanzadeh MT (2020). Effects of single and combined supplementation of dietary probiotic with bovine lactoferrin and Xylooligosaccharide on Hemato-Immunological and Digestive enzymes of silvery-black Porgy Fingerlings. Annals of Animal Science.

[CR65] Morshedi V, Bojarski B, Hamedi S, Torahi H, Hashemi G, Faggio C (2021). Effects of Dietary bovine lactoferrin on growth performance and Immuno-physiological responses of Asian Sea Bass (*Lates calcarifer*) fingerlings. Probiotics and Antimicrobial Proteins.

[CR66] Naiel MAE, Alagawany M, Patra AK, El-Kholy AI, Amer MS, Abd El-Hack ME (2021). Beneficial impacts and health benefits of macroalgae phenolic molecules on fish production. Aquaculture.

[CR67] Naiel MAE, Negm SS, Abd El-hameed SAA, Abdel-Latif HMR (2021). Dietary organic selenium improves growth, serum biochemical indices, immune responses, antioxidative capacity, and modulates transcription of stress-related genes in Nile tilapia reared under sub-optimal temperature. J Therm Biol.

[CR68] Nakajima K-i, Nakamura M, Gao X-D, Kozakai T (2008). Possible involvement of prolactin in the synthesis of lactoferrin in bovine mammary epithelial cells. Biosci Biotechnol Biochem.

[CR69] Nguyen DN, Li Y, Sangild PT, Bering SB, Chatterton DEW (2013). Effects of bovine lactoferrin on the immature porcine intestine. Br J Nutr.

[CR70] Niaz B, Saeed F, Ahmed A, Imran M, Maan AA, Khan MKI, Suleria HAR (2019). Lactoferrin (LF): a natural anti-microbial protein. Int J Food Prop.

[CR71] Pagheh E, Marammazi JG, Agh N, Nouri F, Sepahdari A, Gisbert E, Mozanzadeh MT (2018). Growth performance, hemato-immunological responses, and Digestive enzyme activities in Silvery-Black Porgy (*Sparidentex hasta*) Fed Dietary bovine lactoferrin. Probiotics and Antimicrobial Proteins.

[CR72] Peterson E, Kaur P (2018) Antibiotic resistance mechanisms in Bacteria: Relationships between Resistance Determinants of Antibiotic Producers, environmental Bacteria, and clinical pathogens. Front Microbiol 9. doi:10.3389/fmicb.2018.02928 10.3389/fmicb.2018.02928 PMC6283892 30555448

[CR73] Raghuveer TS, McGuire EM, Martin SM, Wagner BA, Rebouché CJ, Buettner GR, Widness JA (2002). Lactoferrin in the Preterm Infants’ Diet attenuates Iron-Induced Oxidation Products. Pediatr Res.

[CR74] Rahimnejad S, Agh N, Kalbassi M, Khosravi S (2012). Effect of dietary bovine lactoferrin on growth, haematology and non-specific immune response in rainbow trout (*Oncorhynchus mykiss*). Aquac Res.

[CR75] Ren T, Koshio S, Ishikawa M, Yokoyama S, Micheal FR, Uyan O, Tung HT (2007). Influence of dietary vitamin C and bovine lactoferrin on blood chemistry and non-specific immune responses of japanese eel, *Anguilla japonica*. Aquaculture.

[CR76] Sakai M, Otubo T, Atsuta S, Kobayashi M (1993). Enhancement of resistance to bacterial infection in rainbow trout, *Oncorhynchus mykiss* (Walbaum), by oral administration of bovine lactoferrin. J Fish Dis.

[CR77] Sandomirsky B, Galchenko S, Galchenko K (2003). Antioxidative properties of lactoferrin from bovine colostrum before and after its lyophilization. Cryoletters.

[CR78] Sharma D, Shastri S, Sharma P (2017). Role of lactoferrin in neonatal care: a systematic review. J Maternal-Fetal Neonatal Med.

[CR79] Sinha M, Kaushik S, Kaur P, Sharma S, Singh TP (2013) Antimicrobial Lactoferrin Peptides: The Hidden Players in the Protective Function of a Multi-functional Protein. International Journal of Peptides 2013, 390230. doi:10.1155/2013/390230 10.1155/2013/390230 PMC3608178 23554820

[CR80] Siqueiros-Cendón T, Arévalo-Gallegos S, Iglesias-Figueroa BF, García-Montoya IA, Salazar-Martínez J, Rascón-Cruz Q (2014). Immunomodulatory effects of lactoferrin. Acta Pharmacol Sin.

[CR81] Soboleva SE, Sedykh SE, Alinovskaya LI, Buneva VN, Nevinsky GA (2019). Cow milk lactoferrin possesses several Catalytic Activities. Biomolecules.

[CR82] Soliman SA, Emeish WFA, Abdel-Hafeez HH (2022) Lactoferrin improves the immune response and resistance of silver carp, a hematological, light (histochemical and immunohistochemical), fluorescent, and scanning electron microscopic study. Microscopy Research and Technique. doi:10.1002/jemt.24208. n/a(n/a) 10.1002/jemt.24208 35876377

[CR83] Superti F (2020). Lactoferrin from bovine milk: a protective companion for life. Nutrients.

[CR84] Suzuki YA, Lopez V, Lönnerdal B (2005). Lactoferrin. Cell Mol Life Sci.

[CR85] Taherah M (2021) The anti-microbial and immune boosting effects of milk lactoferrin in the fish. World Journal of Pharmaceutical Sciences, 9(7): 45–48. Retrieved from https://www.wjpsonline.com/index.php/wjps/article/view/133

[CR86] Teraguchi S, Wakabayashi H, Kuwata H, Yamauchi K, Tamura Y (2004). Protection against infections by oral lactoferrin: evaluation in animal models. Biometals.

[CR87] Trybek G, Metlerski M, Szumilas K, Aniko-Włodarczyk M, Preuss O, Grocholewicz K, Wiszniewska B (2016). The biological properties of lactoferrin. Cent Eur J Sport Sci Med.

[CR88] Villavicencio A, Rueda MS, Turin CG, Ochoa TJ (2017). Factors affecting lactoferrin concentration in human milk: how much do we know?. Biochem Cell Biol.

[CR89] Vorland LH, Ulvatne H, Andersen J, Haukland HH, Rekdal Ø, Svendsen JS, Gutteberg TJ (1998). Lactoferricin of bovine origin is more active than Lactoferricins of Human, Murine and Caprine Origin. Scand J Infect Dis.

[CR90] Wang T, Secombes CJ (2013). The cytokine networks of adaptive immunity in fish. Fish Shellfish Immunol.

[CR91] Wang Y-D, Chang H-Y, Chen J-Y, Chen J-C (2013). Oral administration of bovine lactoferrin inhibits bacterial infection in tilapia and elevates survival after bacterial infection: an examination of its immune-modulating properties. Aquacult Int.

[CR92] Welker TL, Lim C, Yildirim-Aksoy M, Klesius PH (2007). Growth, immune function, and disease and stress resistance of juvenile Nile tilapia (*Oreochromis niloticus*) fed graded levels of bovine lactoferrin. Aquaculture.

[CR93] Welker TL, Lim C, Yildirim-Aksoy M, Klesius PH (2010). Dietary bovine lactoferrin increases resistance of Juvenile Channel Catfish, *Ictalurus punctatus*, to enteric septicemia. J World Aquaculture Soc.

[CR94] Yan D, Chen D, Shen J, Xiao G, van Wijnen AJ, Im H-J (2013). Bovine lactoferricin is anti-inflammatory and anti-catabolic in human articular cartilage and synovium. J Cell Physiol.

[CR95] Yao X, Bunt C, Cornish J, Quek S-Y, Wen J (2013). Oral delivery of lactoferrin: a review. Int J Pept Res Ther.

[CR96] Yao X, Bunt C, Cornish J, Quek S-Y, Wen J (2015). Oral delivery of bovine lactoferrin using pectin- and Chitosan-Modified Liposomes and solid lipid particles: improvement of Stability of Lactoferrin. Chem Biol Drug Des.

[CR97] Yilmaz S, Yilmaz E, Dawood MAO, Ringø E, Ahmadifar E, Abdel-Latif HMR (2022). Probiotics, prebiotics, and synbiotics used to control vibriosis in fish: a review. Aquaculture.

[CR98] Yokoyama S, Ishikawa M, Koshio S (2019). Dietary bovine lactoferrin enhances defense factors on body surface and anti-parasitic effects against *Neobenedenia girellae* infection, and mitigates low-salinity stress in amberjack (*Seriola dumerili*) juveniles. Aquaculture.

[CR99] Yokoyama S, Koshio S, Takakura N, Oshida K, Ishikawa M, Gallardo-Cigarroa FJ, Teshima S-i (2006). Effect of dietary bovine lactoferrin on growth response, tolerance to air exposure and low salinity stress conditions in orange spotted grouper *Epinephelus coioides*. Aquaculture.

[CR100] Yokoyama S, Koshio S, Takakura N, Oshida K, Ishikawa M, Gallardo-Cigarroa FJ, Teshima S-i (2005). Dietary bovine lactoferrin enhances tolerance to high temperature stress in japanese flounder *Paralichthys olivaceus*. Aquaculture.

